# Living in the shadow of unemployment -an unhealthy life situation: a qualitative study of young people from leaving school until early adult life

**DOI:** 10.1186/s12889-019-8005-5

**Published:** 2019-12-10

**Authors:** Anne Hammarström, Christina Ahlgren

**Affiliations:** 1 Institute of Environmental Medicine, Karolinska Institutet, Stockholm, Sweden; 2 Department of Epidemiology and Global Health, Umeå University, Umeå, Sweden; 3 Department of Community Medicine and Rehabilitation, Umeå University, Umeå, Sweden

## Abstract

**Background:**

Despite the magnitude of youth unemployment there is a lack of studies, which explore the relations between health experiences and labour market position in various contexts. The aim of this paper was to analyse health experiences among young people in NEET (not in education, employment or training) in relation to labour market position from leaving school until early adult life.

**Method:**

The population consists of everyone (six women, eight men) who became unemployed directly after leaving compulsory school in a town in Northern Sweden. Repeated personal interviews were performed from age 16 until age 33. The interviews were analysed using qualitative content analysis.

**Results:**

Health experiences can be viewed as a contextual process, related to the different phases of leaving school, entering the labour market, becoming unemployed and becoming employed. Perceived relief and hope were related to leaving compulsory school, while entering the labour market was related to setbacks and disappointments as well as both health-deteriorating and health-promoting experiences depending on the actual labour market position. Our overarching theme of “Living in the shadow of unemployment – an unhealthy life situation” implies that it is not only the actual situation of being unemployed that is problematic but that the other phases are also coloured by earlier experiences of unemployment .

**Conclusion:**

A focus on young people’s health experiences of transitions from school into the labour market brings a new focus on the importance of macroeconomic influence on social processes and contextualised mechanisms from a life-course perspective.

## Background

Since the great depression around 1930 quantitative research has demonstrated that unemployment is related to deteriorated health, with both mental and somatic symptoms, deteriorated health behaviour and increased mortality [1, 2].

However, there is still a lack of understanding of why these relations exist. What is it in unemployment that increases the risk of ill health? What are the mediating mechanisms? Various models have been suggested in order to explain the relationships between unemployment and ill health. Six main models can be identified. The *economic deprivation model* suggests that the financially strained situation of the unemployed is the actual cause of ill health [3]. The *stress model* implies that unemployment is a chronic stressor with negative impact on health status [4]. The *model of latent functions* was developed by Marie Jahoda [5] based on her research about what needs, besides the economic ones, a good job should fulfil. These needs are called “latent functions” and they imply that employment should give:

* a time structure of the day
* regularly shared experiences and contacts with others
* goals and purposes that transcend those of the employed
* personal status and identity
* enforced activity

The model implies that unemployment causes ill health due to the absence of these five needs (or functions) which (to a smaller or larger extent) are provided by a job.

The *control model* states that the potential to have control over one’s life is crucial for health and well-being. The model has been developed in a variety of theoretical traditions, and empirical research has shown the importance for health of maintaining control during unemployment [6]. The *work involvement model* means that low work involvement during unemployment is protective of one’s health status [7] while the *lack of support model* implies that unemployment is related to deteriorated health due to increased social isolation [7].

A quantitative testing of these six models shows that the capacity of the models to explain the connection between unemployment and ill health varied [8]. The model of latent functions was most successful, followed by the economic deprivation model. The social support and the control models were also fairly good. The work involvement scale and the stress model demonstrated the smallest explanatory power.

Even though the theoretical development and testing of models is a step forward for understanding why unemployment is related to deteriorated health, quantitative methods are based on prefixed answer alternatives, leaving no space for new understandings to be discovered. In order for research to develop knowledge about a poorly understood phenomena, qualitative methods are needed. The results from qualitative research can later on can be tested with quantitative methods to draw conclusions about cause and effect. But there is an almost total lack of inductive qualitative research about health experiences among those who are unemployed. Some of the few qualitative studies in the field are summarized below.

A qualitative study among participants over age 18 from the time of economic recession in Sweden illustrates the importance of gaining better understanding of the experiences of hardship and perceptions of health among those who lose their jobs [9]. Another qualitative study of unemployed persons, aged 18 and above with mental health problems, was performed in Germany [10]. The study illustrates barriers and facilitators for help seeking on three levels: mental health literacy, stigma and discrimination of them as help-seekers and structures and conditions of health care such as miscommunication and GPs lack of interest in mental health problems.

Many young people, who do not have jobs, are not officially registered as unemployed. The concept of NEET – not in employment, education or training – has been increasingly used in order to define a group of mostly young people who are disengaged from the labour market [11]. This group is difficult to reach in both quantitative and in qualitative studies and thus there is a need for more knowledge about their experiences and life situation [12].

A qualitative study from Sweden about health experiences of young people in NEET demonstrates the importance of contextualisation of health experiences [13]. Health was created in relation to others and the participants felt good when closely connected to others. Health was created in relation to severe hardship (like drugs and various forms of violence) as well as in relation to the participants’ ability to adapt and respond to challenges. Thus, health among NEETs was viewed as constructed within the social and cultural context in which the ability to adapt and respond to challenges are crucial. The participants had a diverse background, but still shared common experiences related to feelings of inclusion and exclusion. Thus, health was viewed as created both on an individual and collective level.

The transition from school to work can be influenced by macroeconomic factors such as recession also among young people who are not in NEET. A qualitative study of students [14] showed that the crisis had negative impact on the participants’ mental health by creating feelings of instability as well as difficulties in planning for the future.

The participants clearly experienced disengagement from community participation manifested in feelings of isolation, lack of interest, and distrust. Nevertheless, the respondents described a proactive attitude to deal with the problems they were facing, without giving up their commitment to personal fulfilment.

These qualitative studies demonstrate that open interviews about health experiences among different groups of unemployed can provide a deeper insight into why these health experiences occur as well as in which context they develop. Therefore, qualitative methods are needed in order to analyse such experiences. All research, especially qualitative, must be contextualised in order to increase the trustworthiness and the transferability of the findings.

We have demonstrated in a review that most quantitative research on unemployment and ill health lacks such contextualisation as very few studies have analysed the results separately per subgroup of population in relation to, for example, age and gender [15]. Most empirical and theoretical research on this topic has been performed in adults, in spite of the growing evidence of both the short-term [16–18] and the long-term [19–22] negative health consequences of unemployment early in life. The aim of this paper was to analyse health experiences among young people in NEET in relation to labour market position from leaving school until early adult life.

## Method

### Setting

Interviews for this qualitative study were performed within the Northern Sweden Cohort [23]. The cohort was created in 1981 in a medium-sized (with about 70,000 inhabitants) industrial town in Northern Sweden. The town is representative of medium-sized industrial towns in Sweden as regards the sociodemographic factors and labour market conditions. The cohort has also been shown to be representative of the country as a whole in relation to socio-demographics and socioeconomic factors as well as in relation to health status and health behaviour [23]. The labour market is dominated by manufacturing and mineral extraction, with a steel company and a large harbour. Other major employers are the public sector and a technical university. At the time of the interviews, unemployment in the town was twice as high rate as in the rest of Sweden and there was more workforce immigration from Finland. The social democrats had ruled the area for decades together with smaller socialist parties.

In order to tackle the growing rate of youth unemployment the Swedish government introduced active labour market policy measures for young people from the beginning of the 1980s. These measures included educational and vocational activities directed towards unemployed young people with the objective that no one aged 18 years or below should be outside education or employment. Subsidised employment, with work tasks that did not compete with regular jobs, was the most common labour market measure for young people at the time when the cohort participants were young. Labour market policy measures for people below 18 years were financed by the state with study assistance instead of salary and were intended to guarantee activity for 8 h a day for 40 weeks for unemployed in the age-group 16–18 years. In 1982, the time for reimbursement for these programmes was somewhat increased beyond 40 weeks and in 1984, a law was introduced stating that unemployed aged 18–19 should participate in labour market measures at least 4 h a day and for a minimum wage ([16] pp 4). This meant that, by international standards, Sweden had a very active labour market policy aimed at young people during the 1980s. For the participants in our study, these active labour market policy programmes meant that they did not become permanently long-term unemployed but rather moved between unemployment, various labour market programmes and short periods of temporary employment. However, lack of resources due to regional differences in number of unemployed could have been the reason why cohort participants were not contacted by the youth centre and instead became unemployed.

### Design and ethics

A qualitative study design was chosen. Repeated interviews were conducted with young women and men aged from 16 to about 30, concerning their experiences of work and unemployment and associated health experiences. The interviews were analysed with qualitative content analysis.

### Participants

The interviews were conducted with a subsample of participants in the Northern Swedish Cohort – a prospective longitudinal cohort study. The cohort consists of all pupils (*n* = 1083) in their last year of compulsory school (age 16) in all nine schools in a medium-sized town in Northern Sweden [23]. The cohort has been followed over time with questionnaires about their health status and life circumstances. A subsample of the cohort has been followed with personal interviews at the age intervals 16–17, 21–23 and 28–33 years. This subsample constitutes the base for this study.

The subsample consists of all cohort participants who became unemployed directly after compulsory school (six women and eight men). They had all left school at the age of 16 (some of them without full grades) and were all of working-class background. They were interviewed two to four times per individual during a time period from age 16 until age 33 (between 1981 and 1998).

### Data collection

Individual audio-recorded structured interviews were performed by the PI (principle investigator) of the cohort (AH, Anne Hammarström), a medical doctor and specialist in social medicine. The interviews dealt with daily activities in various spheres, possibilities of influencing one’s life, dreams for the future and experiences of health and unemployment/work (see Interview Guide in the Additional file 1). The interviews were performed in the informant’s home or at the PI’s workplace and lasted for about 1 to 1½ hours. The same informants were interviewed two to four times at different ages. In total 37 interviews constituted the base for the analysis. Health at age 30 was collected both from the interviews and from the informants’ answers to questions about health in a questionnaire to the whole cohort.

### Data analysis

The interviews were analysed using qualitative content analysis as described by Graneheim and Lundman [24]).Qualitative content analysis is used in systematic analysis of verbal communication [25] and is useful in analysing people’s experiences and reflexions [26]. Furthermore, it is a suitable method for focusing on similarities and differences in the material [24].

The interviews were read through several times by both authors to get a sense of the whole. Thereafter, the analysis was performed in several steps using the software package Open Code [27]. In each step CA (Christina Ahlgren) did a preliminary analysis, which then was discussed between the authors. In cases of disagreement, we reread the transcripts in order to catch the initial meaning and adjusted the interpretations of codes and categories’ until agreement was reached. In the first step of the analysis, the text was divided into meaning units, each comprising several words, sentences, or paragraphs containing aspects of health during employment and unemployment. After that the meaning units were labelled with codes and sorted into content areas. A content area is a rough structure of content that can be identified with little interpretation. The content areas in this study were parts of the text dealing with the informants’ experiences of unemployment and employment periods at different ages, which resulted in four phases defined as leaving the school phase, entering the labour market phase, the unemployment phase and the employment phase. Thirdly, codes with similar meanings were summed into categories with subcategories. Categories can be seen as manifest interpretations of the text and should answer the question “What?” [25]. Categories can include subcategories. In this study categories and subcategories were formed through interpretations of codes representing the informants’ experiences of unemployment and employment and associated aspects of health. Fourthly, a theme was suggested, through interpretation of the underlying meaning of health and health problems related to the experiences described in the categories. A theme could be seen as an interpretation of the latent content of the text and answers the question “How?” [24]. Sub-categories, categories and a theme are presented in Table 1.

**Table 1:** The table shows the inter-relationship between the sub-categories, the categories and the theme

Sub-categories	Categories	Theme
+ Free from school	Perceiving relieve and hope	Living in the shadow of unemployment – un unhealthy life situation
+ Getting a job		
+ Earn own money		
+ Dreams about the future		
+ Feel fine		
- Job applications rejected	Noticing set-backs and disappointment	
- No money		
- Dependency		
- Distrust		
- Disappointment		
- Passive life-style	Perceiving hopelessness and resignation	
- Lack of money		
- Deteriorating self-confidence		
- Intense smoking and drinking		
- Bad eating habits		
- Stress-related health symptoms		
- Mental health symptoms		
- Negative mood		
+ Required to help mothers	Perceiving caring responsibilities	
+ Structuring the day		
+ Reduced night-life drinking		
+ A pause from working life demands		
+ Social acceptance		
+ Sense of being needed	Perceiving hope and social worthiness	
+ Being trusted and given responsibilities		
+ Structuring of the day		
+ Feelings of pride		
+ Restrict late nights drinking		
-Experiencing job insecurity	Accepting adverse work situations	
-Perception of stress		
-Work-related injuries and illness		

+ denotes experiences associated with positive health, − experiences associated with deteriorated health

In order to further, achieve trustworthiness the two researchers from different fields of expertise (family medicine/social medicine and physiotherapy) worked in parallel during the whole analysis process and discussed the findings until agreement was reached. This is in accordance with the process of triangulation [24, 28]. The findings were also discussed with other researchers in the field and found trustworthy.

## Results

**Fig. 1:**
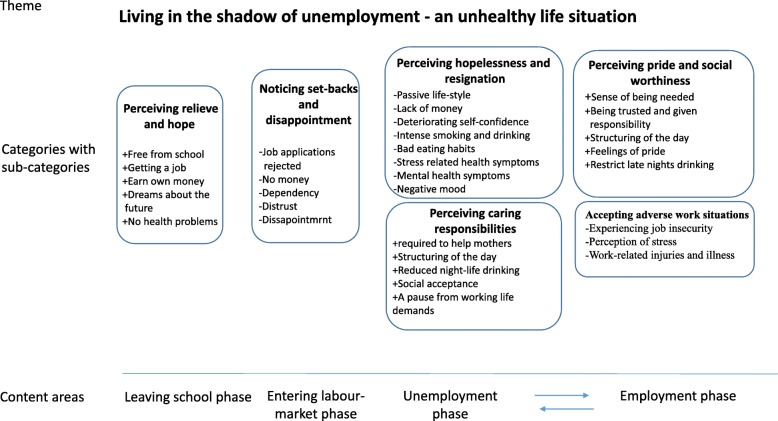
The figure describes a tentative process of the relationship between the informants’ experiences and feelings (illustrated in sub-categories and categories) during phases of unemployment and employment from leaving school at 16 year of age until 30 years of age and how their experiences could be related to positive health symptoms and deteriorating health. + denotes experiences or feelings associated with positive health -denotes experiences or feelings associated with deteriorating health

The analysis resulted in a tentative process of the development of positive health or health problems by interpreting the informant’s feelings and experiences of unemployment and employment from leaving school at age 16 to adulthood at age 32. The process is described in the text and in Fig. 1.

The informant’s experiences during each phase are described in six categories, distributed over the four phases. Leaving the school phase is characterised by the category “Perceiving relief and hope”. Entering the labour market phase by the category “Noticing setbacks and disappointments”. The unemployment phase by “Perceiving hopelessness and resignation” and “Perceiving caring responsibilities” and the employment phase by “Perceiving pride and social worthiness”, and “Accepting adverse work situations”. Each category includes subcategories. The theme “Living in the shadow of unemployment – an unhealthy life situation” describes a tentative process between the categories and experiences of health

### “Living in the shadow of unemployment – an unhealthy life situation”

The theme is built upon the interviewees’ expressions of experiences and feelings during employment and unemployment phases and our interpretations of how this could be associated with health and health problems. Experiences during unemployment phases were often expressed in terms of passivity, resignation and poverty and were linked to feelings of deteriorated mental health, i.e. dysphoria, depression and low self-confidence. Experiences and feelings counter-balancing deteriorated health during unemployment phases were mostly expressed by women. Employment phases, on the other hand, were mostly mentioned in relation to positive health experiences, i.e. being trusted and a sense of being needed. However, the short-term contracts with a constant risk of being unemployed again induced stress. The fear of unemployment also made them accept non-optimal work environments, although they could affect future health.

#### Perceiving relief and hope

This category corresponds to leaving the school phase and embraces the first half year after leaving compulsory school at 16 years of age and is dominated by the informants’ positive thoughts directly after leaving compulsory school. They felt relieved at quitting the school they did not like or even hated during the last school years. The reasons for the dislike were multifactorial and included “*teachers are unfair*”, “*school is of no use in real life*”, or “*I was bullied*”. However, some participants also said that school was okay, but that they were “*bored*”. They expressed a desire to become mature by getting a job and earning one’s own money, which also meant being less dependent on parents. Their dreams about the future included both specific occupations and standards of living, for example being able to afford to buy a car of one’s own, or a motor bike, to travel abroad and to buy clothes. The occupations they dreamt of were partly gendered, in that young men dreamt of driving their own lorry or starting a delivery firm while young girls dreamt of working in caring occupations or in shops.

Health issues were not on their agenda, and the feelings expressed were primarily positive.

#### Noticing setbacks and disappointments

Leaving the school phase gradually passed into Entering the labour market phase at about 17 years of age, when the feelings of an extended holiday faded away and the informants still were out of work.

The category describes the situation most of them faced when their dreams about life after quitting school were slowly crushed. They told about the disappointment when job applications were rejected repeatedly with the notification that they were too young and had no work experience. They had lot of leisure time but no money, which made them dependent on parents’ economic contributions.

Due to the prescribed social/labour market policy, the informants in our study became dependent on employment officers at the youth centre to get work. However, the informants expressed distrust against them and said that the only information they gave was that there were no jobs available unless they were prepared to move south. Neither the 16-year-old participants nor their parents appreciated this advice, because it meant sending a 16-year-old son or daughter to a large city almost 500 miles away. When job training courses were offered via the youth centre, some of the informants found them very positive, while others rejected them as being low-paid and not real jobs. Some of the informants had succeeded by themselves in getting jobs for short periods, for example in workplaces they had come into contact with during trainee jobs when still at school.

In this phase as well, all informants assured that they were in good health, although some suffered from health problems, for example congenital hip disease, allergy and headache. These were not considered as health problems, but just something they had. On the other hand, they expressed feelings of disappointment when they talked about the conflicting situation between their hopes for a job and the setbacks with repeated rejections of job applications.

#### Perceiving hopelessness and resignation

This category describe the informants’ experiences during unemployment. However, neither the unemployment nor the employment situation was permanent over the years. Instead they altered between the unemployment phase and the employment phase and the time spent in each phase varied between individuals. Although informants perceived the first months of unemployment as an extended school holiday, when they were free to do whatever they liked, it became boring after a while. With time and still alternating between short temporary employment and unemployment, it was experienced as extremely discouraging. The unemployment situation led to a very passive lifestyle among informants of all ages, although somewhat differently expressed with age. The common response to the question What do you do during the days? was *“I do nothing*”. Among the young informants still living with their parents, it led to a reversed structure of the day. Many of them slept long in the mornings to make time pass, “*There’s no point getting up in the morning, because there is nothing to do”* and stayed out late in evenings, drinking beer, and carrying on with friends. This resulted in awakening late next day and the vicious circle continued.

Informants who had children or a partner with a job had to get up in the morning, but idleness during the days was experienced as very hard. Despite having a lot of time they found it difficult to find the energy to engage in activities. Much time was spent watching television.

“*You don’t do anything when you’re unemployed. I was restless. When I had money I could drive around in the car and busy myself with that, and a bit of everything. I looked for work, but I didn’t really do anything useful, if I put it like that.*”

“*Unemployment periods are extremely difficult. … The days are endless. … I sometimes help my sister with her children, when she is at school*.”

Their social contacts were solely with other unemployed friends, while those who were employed were busy at work. This made our participants feel even more outside the labour market.

Lack of money and small financial resources were experienced by all informants during the phases of unemployment, although it was more often emphasised in the interviews with men. They talked about their wish to get a driver’s licence and buy a car. Women’s wishes concerned having an apartment of their own and being able to buy nice clothes.

“*The most negative thing about unemployment is the shortage of money. You can’t do anything and when you start a new job, you have to wait a month for the salary”.*

Several men said that they had missed jobs due to lack of money to pass a driving test.

“*They needed people and I could have gone on working there if I’d had a licence to drive a heavy lorry*.”

Their poor financial situation restricted their possibilities to make use of the spare time which they had too much of. Some participants were entitled to unemployment benefit for a while, but felt stressed about finding a new job during this time. Others were dependent on parents or on social welfare, which was experienced as a defeat.

One of the men admitted that he had stolen when he was out of money. However, he was caught, which ended his criminal career, but this also ruined his economy further, due to the fines he had to pay.

Deteriorating self-confidence and feelings of low worth were expressed in relation to rejections of job applications even several years after leaving school and when employment officers told them that there were no jobs available for them because they lacked formal education. This was even more apparent with longer periods in unemployment, which is illustrated with quotations from a man and a woman in their early twenties.

“*You feel worthless when unemployed. You become work-shy*.”

“*Being unemployed gives you low self-confidence. I feel that I know nothing and am not capable of anything.*”

Feelings of irritation and aggression were experienced and handled differently. Some turned to more excessive drinking to hide their low self-confidence while others were more depressed.

“*You don’t get self-confidence from being unemployed. You do nothing but sleep all day and hang around. I was quite aggressive when unemployed*.”

The repeated rejections of job applications led to resignation in some of the informants, who thought there was no point in keeping on trying to find a job, which is illustrated with quotations from a young man and a young woman.

“*There’s no point applying for a job, because there are no jobs*.”

“*I find it tiresome to be unemployed, but you get used to it and become more and more apathetic. You want to do something, and then you realise that there’s no point in trying, that’s what makes it tiresome*.”

*“I think it’s no use exerting yourself, you are nothing. You get fed up being out of money*.”

Intense smoking and drinking was common among both young men and women during unemployment. In order to counteract the boring passivity during the days, they spent evenings and nights out with friends. This was often accompanied by drinking alcoholic beverages, and some told of excessive drinking. By being in such situations some of the men had been involved in fights and been physically abused, which had given one of them persistent harm, with psychological problems and memory loss. Other men had been involved in criminality and had been accused of theft and assault and drinking and driving, which had resulted in accidents and fines.

Bad eating habits were common when unemployed. The informants told about not having breakfast or lunch and only coffee and cakes during the day and fast food in the evenings. Reasons cited for bad eating habits were passivity and resignation.

“*After a period you didn’t bother to cook at home. You didn’t bother to go out to buy food*.”

One young woman was on a constant diet, because she had been bullied in school for being too fat. She also continued dieting as an adult and this affected her little daughter, who was given the same diet food.

Stress-related health symptoms during unemployment phases took physical expressions as stomach-ache and headache. In addition mental health symptoms such as anxiety depression and sleeping problems were often experienced as well as negative moods such as restlessness, aggressiveness, dysphoria and low self-confidence. One young woman talked of several health-damaging feelings during unemployment.

“*Unemployment is very stressful. I feel restless and depressed when I can’t get a job and I get stomach pains and sleeping problems*.”

#### Perceiving caring responsibilities

Gendered caring responsibilities were a recurrent subcategory during the whole follow-up period and seemed to counterbalance some of the negative experiences of unemployment. Although the young women also talked about being passive during unemployment, many of them said that their parents required them to do tasks at home when unemployed. For example, they were expected to help their mothers to take care of younger siblings or to clean the house, something none of the young men talked about.

Several of the women became pregnant in their late teens and thus they found a purpose in life. Being responsible for children and family gave them a more structured day and reduced their patterns of night-life drinking. Also when growing older women told about becoming a mother as a positive interruption of unemployment. To be on parental leave was experienced to give a respectable position in society in contrast to being unemployed.

“*I was out drinking and smoking a lot the last year at school. Nowadays I hardly drink any alcohol at all, I have to take care of the children*.”

Unemployment could also be experienced as a relief from strenuous work tasks and a possibility to stay at home with the child, as this woman on parental leave said:.

“*Earlier when unemployed I felt irritated, which I’m not now. Instead I find it a relief not to have to leave my son in day care.*”

Although men did not talk about taking care of children, one man said he had to support his mother financially and therefore had to take on all kinds of jobs he was offered.

#### Perceiving pride and social worthiness

The category includes the informants’ experiences during periods of employment (employment phases). With time most of the participants managed to find a job on shorter or longer contract basis. Having a job could refer to participation in labour market measures arranged by the youth centre, long- or short-term contracts on the labour market or work tasks arranged by relatives. Mainly positive feelings were expressed about getting employed, although negative feelings were also mentioned.

Having a job was experienced as a sense of being needed, of being a valuable citizen and doing something valuable. This was strengthened by a sense of belonging to a team at the workplace and accompanied with feelings of happiness and pride. This was acknowledged in informants of all ages.

“*The best thing about having a job is being with nice workmates*.”

“*The best thing about the job at the steel company is the lads. They are so cool.*” To be treated with respect and expected to be a capable worker was a positive experience which made them feel needed and made them endeavour to do their best.

“*It feels nice, when they come and give you a new task which they think you can manage. It’s nice that they trust you to pull it off so you exert yourself to do it well*.”

A woman at age 32, working as an assistant nurse in a home for the elderly, expressed her responsibility for the care recipients:

“*We have this discussion about cleaning the rooms or not. We take care of everything and have great responsibility, both caring and cleaning. But we are there for the residents, aren’t we*?”

A feeling of being trusted and given responsibility was conveyed by some of the 30-year-old informants who had been offered jobs. They had either approached the employers earlier and asked for a job or had previously had temporary employment at the workplace. Being offered employment without having applied for it was accompanied by a sense of having a reputation for being competent, which made them extremely proud.

“*I didn’t have to apply for this position. They had heard of me and came and asked me to work there*.”

*“I had contacts from before and phoned and introduced myself. I was offered lots of jobs. I think it’s up to you. No jobs comes in your mailbox, you have to get involved.*”

Having a job meant a structuring of the day, getting up in the morning and working 5 days a week. Although some of the young informants found it tiresome it was still seen as part of maturing and taking responsibility. In their early twenties the participants still felt the need for a job to structure their days:

“*A job means that you get up in the morning*.”

Having a job also meant that leisure time was felt to be more valuable.

“*When you are unemployed, you have a lot of time, but you don’t do anything. When you have a job, you do something meaningful during the days and also have time to do something in the evenings*.”

Having a job was mostly associated with positive feelings. The interviewees’ expressed feelings of pride and being a respectable citizen, someone who took responsibility and was trusted. In order to manage the job, they had to restrict their late nights out drinking and instead living the life of an ordinary “Svensson”. Being in employment phases seemed to be health-promoting. This is understood from the answers “*I’m fine, could not be better*”, “*I have a good life*” and” *I feel like a prince*”, which were given by informants with jobs to the question of how they perceived their health.

Social support seemed to be crucial for longer times in employment phases, in terms of parents’ help to find jobs or persuading the participants to study, or partners’ help to create a secure social situation.

#### Accepting adverse work situations

Many informants also described negative experiences of their labour market situation and had health problems related to their jobs. Informants who worked on short-term contracts for long periods experienced job insecurity*.* The joy they felt from having a job was accompanied by a perception of stress, as they knew that they would soon have to quit. The insecurity in a temporary job made some informants feel that they had to abstain from complaints, even justified ones, in order to make a good impression and be eligible for a new employment.

“*I have this job and love it, everybody is so nice to me. I can’t continue as the ordinary staff want full-time employment. It feels very sad*.”

“*I don’t know but it feels harsh, you have been there for six months and then … just leave*.”

Those who still were in temporary employment when approaching 25 years and had family responsibilities perceived the future as very uncertain. Although the temporary jobs gave a salary for the moment and contacts with future employers, they worried about their financial situation in the future. They had to move between workplaces and repeatedly adopt to new work teams, which contributed to the feelings of insecurity. To accept some of the jobs offered, they had to move away from family and friends.

“*You get a chance to get a job in a company, but you never know how long you’re allowed to be there and what next month will be like. This makes me anxious, which is not good.*”

Health problems in relation to jobs also included workplace injuries and illnesses due to physically heavy work tasks and working in other unhealthy environments. By being eager to take on all kinds of jobs the participants accepted unhealthy and dangerous work situations or set security aside. For example, a young man who took on all jobs he was offered was hit by an overhead crane during work at a steel company. This damaged his shoulder and caused him persistent pain which in turn made it harder for him to find another job in the future. Similarly, a woman avoided unemployment by engaging in shoeing horses. She was kicked in the shoulder by a horse and became partly impaired for a long period of time. Another example was a young man with asthma from childhood, who experienced a worsening of his symptoms when he worked in a very dusty environment sorting newspapers for recycling.

## Discussion

This qualitative study showed that the health experiences among young people in NEET can be viewed as a contextual process, related to the different phases of leaving school, entering the labour market, becoming unemployed and becoming employed. Perceived relief and hope were related to leaving compulsory school, while entering the labour market was related to setbacks and disappointments as well as both health-deteriorating and health-promoting experiences depending on the actual labour market position. Our overarching theme of “Living in the shadow of unemployment – an unhealthy life situation” implies that it is not only the actual situation as unemployed that is problematic but that the other phases are also coloured by earlier experiences of unemployment. The results should be interpreted in the light of the Nordic welfare system, with extensive active labour market policies during the period when the cohort members were young. These policies contributed to keeping the levels of unemployment on a quite low level but still the labour market measures were not enough to prevent everyone from unemployment during certain periods. In the case of this study, the youth unemployment rate of the county was about 13% as compared to 4% in Stockholm [16 pp 6].

In our inductive analysis, we identified various health experiences among young people in NEET. Periods of unemployment were related to stress related symptoms (such as stomach and headache) as well as to mental health symptoms, such as restlessness, aggressiveness, anxiety, dysphoria, depressiveness and sleeping problems and deteriorating health habits. The participants described a vicious circle, with symptoms adding to each other and to intense drinking. An interesting gendered finding was that while both boys and girls described intensified drinking related to unemployment only girls described that when becoming a parent their caring responsibilities for the child made it impossible for them to be out drinking. These findings provide a deeper understanding of our earlier publications of quantitative analyses of alcohol consumption among unemployed youths, were we found that unemployment generally increased the risk of drinking, but not among young women who had children [29].

In this inductive approach, we also identified other experiences which we interpret as possible pathways or mechanisms between the health experiences and the labour market experiences. These mechanisms could partly be related to the models mentioned in the Introduction, mainly the economic deprivation model (no money, being dependent), the model of latent functions (passive lifestyle, deteriorated self-confidence) and the stress model (stress, stress-related health symptoms). Other mechanisms were not related to these models such as being dependent, experiencing distrust, feeling disappointed, resignation, gendered caring responsibilities and deteriorated health behaviour (intense drinking, bad eating habits). In this regard our findings are in accordance with one of the few qualitative studies in the field [30]. The “traditional” models for explaining health effects of unemployment do not fully account for people’s own interpretation and experiences. Thus, our study contributes new understandings about why unemployment is related to ill health among young people.

In earlier research, we have identified a “scarring” mechanism of early unemployment [19–22]. In this context, scarring means that, while youth unemployment has well-known direct effects on health (“wounds”), the wounds remain as scars in adult age (measured in relation to blood pressure and various measures of mental health). Could the mechanisms identified in this study explain scarring? May feelings related to unemployment in young age – such as disappointment, dependence, distrust and resignation – become embedded in the mind and in the patterns of reactions so that those who have been early unemployed respond to stressful life-events later in life with similar reactions and feelings, instead of with an active engagement against injustice and disappointments in life? Feelings of disappointment, dependence, distrust and resignation are closely embedded in depressive states and may be a key to our understanding of unemployment scarring.

We also identified mechanism for improved health during periods of employment. One of these mechanisms was related to the model of latent functions (structuring the day), while several were partly related to the model (being needed, being trusted and given responsibility and feelings of pride). Improved health behaviour was another positive mechanism during employment.

The participants experienced pride and worthiness related to getting employment. Pride could be viewed as a dimension of inner strength, well-being and thus as a positive aspect of health [31]. In addition, in order to manage the job they had to restrict their late nights out drinking.

However, the employment phase during a context of living in the shadow of unemployment was also hazardous to health. The participants, who all experienced a shorter or longer period of NEET, entered the labour market from a marginalised position. Thus, the kinds of jobs and employment they received were on the margin of the labour market where contracts are insecure and short-term. The participants described stress, injuries and adverse working conditions, which are well-known adverse health consequences of precarious employment [18]. Earlier quantitative research has demonstrated the significance of high levels of unemployment in society not only for the unemployed [22] but also for young people in work as well as in studies [32]. Thus, the positive effects on health of finding employment are threatened by macroeconomic influences such as recession. Then, the work environment is deteriorated due to increased stress in several sectors in society, such as health care, social services etc. The work-related demands increase on these women-dominated workplaces due to deteriorated health in the population and increased need for social security benefits for unemployed, while at the same time there is a reduction in the number of employees, resulting in increasing job strain among those who still are employed [32]. In addition, other macroeconomic influences such as the increasingly flexible labour market may hamper the positive experiences of getting employed, as insecure employment contracts are becoming increasingly common, especially among young people. There is increasing evidence of the negative health impact of temporary employment [33].

Our findings emphasise the need to analyse the labour market situation of young people as a contextualised continuum, rather than as a dichotomy, from leaving school to entering the labour market. Even when young people have entered the labour market, they move between the phases of unemployment and training or employment due to the lack of available jobs in society. The importance of bringing a life-course perspective into the research is in accordance with research by Dooley and Prause [34] who (focusing mainly on adults) have highlighted the need to conceive employment status as a continuum, from adequate employment to inadequate employment and to unemployment. Another well-known theoretician within the field of unemployment and health, Douglas Ezzy [35], also proposed a middle-range theory of status passage in order to deal with the inadequate temporal aspects of earlier research. He explained changes in mental health among adults during unemployment derived from identity theory. Inspired by a middle-ranged theory developed by Ezzy [35], Giuntoli et al. [30] have performed a qualitative study among a group of wide age range who had all lost their jobs. Despite the different sample in their study as compared to ours, the mechanisms they identified were similar (unemployment and welfare stigma) or the same as in our study (financial strain, difficulties in finding a new job, personal identity crises, loss of time structure). Giuntoli et al. [30] propose a middle-range theory, which suggests that the effects of employment transitions on people’s mental health are linked with people’s experiences of these passages. This is similar to the results from our inductive qualitative analyses.

In one of the few qualitative studies within the field, Simmons et al. [36] conclude that the broader social structures have a significant impact on becoming and or remaining in NEET. These processes are deeply integrated into a framework of “agency within structures”. In earlier research on the young men who are part of this study we have developed a model of agency within structures in relation to health experiences [37]. There we interpret our findings as constructions of masculinities within certain structures, in relation to choices, habitus and healthy practices. Agency is surely important in relation to the context of this study, for example from the identified mechanisms of wanting a job, searching for jobs in the first phase of entering the labour market, to resignation due to experiences of unemployment (lack of agency). Like us, Simmons et al. [36] found that repeated negative labour market experiences had a de-motivating effect on job-searching among the young people. As a result of continued failure to secure employment, the young people became disillusioned. Again, dissolution may help explain the long-term scarring of yearly unemployed as discussed above.

### On the methods

The strength of this study is the repeated interviews with young people from 16 to 32 years of age and with the same focus. This gives us unique and rich data together with insight into how the young informants’ experiences of health varied with contextual factors, e.g. job/unemployment and changes in their family situation.

In qualitative content analysis one aspect of trustworthiness is dependability. Dependability concerns the stability of data over time and the researchers’ decisions during the analysis process [24]. In our study the same researcher (AH) did all the interviews and used the same interview guide. This might raise questions about dependability but could also be seen as a strength because a trustful relation between researcher and interviewees was created and might have made it easier to talk about sensitive matters for example drug use and criminal activities.

In our study, the interviewees were disfavoured young people with early unemployment due to interrupted school attendance, and the context in which data were sampled was the working class in an industry-dominated city in northern Sweden. Therefore, the findings might be related to similar groups in similar contexts. However, regulations for unemployment insurance greatly affect the possibilities for job training, subsidised employments and so on and have to be considered when generalising.

## Conclusions

A focus on young people’s health experiences of transitions from school into the labour market brings a new focus on the importance of macroeconomic influence on social processes and contextualised mechanisms from a life-course perspective.

## Supplementary information

**Additional file 1.** Interview guide.

## Data Availability

Data are not freely available. The Swedish Data Protection Act (1998:204) does not permit sensitive data on humans (like in our interviews) to be freely shared. After ethical approval the anonymous data set could be obtained on request to Umeå University after their secrecy examination.

## Abbreviations

AH: Anne Hammarström
CA: Christina Ahlgren
NEET: Not in education, employment or training
PI: Principle investigator
