# Supplementary material for: Living in the shadow of unemployment -an unhealthy life situation: a qualitative study of young people from leaving school until early adult life
Source: BMC Public Health. 2019 Dec 10;19:1661. doi: 10.1186/s12889-019-8005-5 (PMC6905008; doi:10.1186/s12889-019-8005-5)
Supplement: Supplementary file 1 — Additional file 1. Interview guide. [file 12889_2019_8005_MOESM1_ESM.docx]

**Interview guide (modified according to age)**

Please tell me what you have done since we met last time?

How did you get the job/labour market measure/studies? Via job centre or did you arrange it yourself?

How did you like what you did?

(If relevant: Why did you leave your job/studies etc.?)

Please tell me what you do now? Tell me about the job/studies etc. If unemployed; tell about your time as unemployed? What do you do?

Please, tell me about how you like or dislike what you do?

How do you manage your finances?

What do you do on your spare time? How do you like it?

What about your use of alcohol? Narcotics?

Please tell me what you did yesterday; from you woke up until bedtime?

Did you do what you wanted to do? What would you have liked to do?

Which were the obstacles for doing what you wanted to do?

Please, tell me about your health status, how do you feel like? What makes you feel good? What makes you feel bad?

Have you had any problems like stomach ache, head pain, sleeping difficulties, concentration difficulties, feeling depressed? Tell me about them. What makes them get better? Or worse?

Do you use any medication? Tell me about them.

How do you feel now compared to when you were (unemployed, employed)?

What are your future plans?

Do you believe that you can do what you want to do?

How do you feel when you think about the future?
